# Dog Ownership during Pregnancy, Maternal Activity, and Obesity: A Cross-Sectional Study

**DOI:** 10.1371/journal.pone.0031315

**Published:** 2012-02-15

**Authors:** Carri Westgarth, Jihong Liu, Jon Heron, Andrew R. Ness, Peter Bundred, Rosalind M. Gaskell, Alexander J. German, Sandra McCune, Susan Dawson

**Affiliations:** 1 Faculty of Health and Life Sciences, Institute of Infection and Global Health, University of Liverpool, Neston, Cheshire, United Kingdom; 2 Arnold School of Public Health, University of South Carolina, Columbia, South Carolina, United States of America; 3 School of Social and Community Medicine, University of Bristol, Bristol, United Kingdom; 4 School of Oral and Dental Sciences, University of Bristol, Bristol, United Kingdom; 5 Faculty of Health and Life Sciences, Institute of Psychology, Health and Society, University of Liverpool, Brownlow Hill, Liverpool, United Kingdom; 6 Faculty of Health and Life Sciences, Institute of Ageing and Chronic Disease, University of Liverpool, Neston, Cheshire, United Kingdom; 7 WALTHAM® Centre for Pet Nutrition, Waltham-on-the-Wolds, Melton Mowbray, Leicestershire, United Kingdom; UCL Institute of Child Health - University College London, United Kingdom

## Abstract

The Avon Longitudinal Study of Parents and Children (ALSPAC) is an observational study of 14273 UK pregnant singleton mothers in 1990/1991. We examined outcomes of self report of strenuous activity (hours per week) at 18 and 32 weeks of gestation, hours spent in leisure-time physical activities and types, and pre-pregnancy body mass index (BMI); overweight status was defined as pre-pregnancy BMI≥25 and obesity BMI≥30. Pet ownership and activity data were reported for 11,466 mothers. Twenty-five percent of mothers owned at least one dog. There was a positive relationship between participation in activity at least once a week and dog ownership (at 18 weeks, Odds ratio 1.27, 95% confidence interval 1.11–1.44, P<0.001). Dog owners were 50% more likely to achieve the recommended 3 hours activity per week, equivalent to 30 minutes per day, most days of the week (1.53, 1.35–1.72, P<0.001). Dog owners were also more likely to participate in brisk walking activity than those who did not have a dog (compared to no brisk walking 2–6 hrs per week 1.43, 1.23 to 1.67, P<0.001; 7+ hrs per week 1.80, 1.43 to 2.27, P<0.001). However, no association was found with any other types of activities and there was no association between dog ownership and weight status. During the time period studied, pregnant women who had dogs were more active, through walking, than those who did not own dogs. As walking is a low-risk exercise, participation of pregnant women in dog walking activities may be a useful context to investigate as part of a broader strategy to improve activity levels in pregnant women.

## Introduction

Maternal obesity before and during pregnancy has adverse outcomes for both mother and child. There is evidence that women may be unaware of these effects and that weight management information and advice from professionals is not always received or assimilated [1]. This has led to recent direction for clinicians to advise pregnant women to manage their weight and exercise [2] and the development of new guidelines in the UK [3].

Pre-pregnancy BMI is a major determinant of pregnancy outcome, with maternal obesity associated linearly with higher risk of many complications [4]. High gestational weight gain by the mother during pregnancy can be associated with adverse pregnancy outcomes and can lead to post-partum weight retention, which may have further repercussions for following pregnancies [4]. Weight status during pregnancy may also have implications for future obesity in the child; parental obesity pre-pregnancy has been shown to be a risk factor for rapid weight gain from 3–5 years [5], and obesity at 7 years [6].

The most successful interventions to tackle maternal obesity would be to prevent the development of obesity before the reproductive years, but this is proving difficult given the upward trend in general obesity, including adolescent girls [4]. Since obesity is caused by an imbalance between energy intake and energy expenditure, lifestyle interventions including increased physical activity are popular treatment strategies in non-pregnant individuals. However, in pregnant individuals dietary energy restriction may be more risky to the foetus than mild to moderate exercise [3].

Generally recommended exercises during pregnancy (in the absence of complications) include walking, hiking, jogging/running, aerobic dance, swimming, cycling, rowing, cross-country skiing and dancing; many women use walking as their primary means of exercise during pregnancy [7]. To date, no studies have reported adverse effects of exercise during pregnancy on the preterm births after 18 weeks of gestation and the preterm risks seem to be limited to high impact exercise [8]. Indeed, some studies of recreational physical activity during pregnancy have shown a reduced risk of pre-term birth, but this may partly be due to confounding with social circumstances that favour positive health behaviours and promote physical activity [9]. Exercise during pregnancy may reduce insulin resistance [10], thus potentially reducing foetus adiposity and birth weight, in particular in large for gestation-age infants [4].The risk of gestational diabetes may also be reduced by physical activity prior to and during pregnancy [11].

Previous studies into companion animal ownership provide evidence that pets may confer both physiological and psychological health benefits [12], [13], [14], [15], [16]. Physical inactivity is one of the top 10 causes of death and disability in the developed world, due to its effects on conditions such as heart disease, cancers, diabetes, obesity, strokes and hypertension [17]. Dog ownership and dog walking are associated with higher physical activity levels [18], [19], [20], [21], [22], [23], [24], [25] and, after adjusting for confounders, dog owners are reported to be 57% more likely to achieve the recommended level of physical activity than non-owners [19]. Some suggest that dog walking is also associated with decreased weight status [25]. However, studies from the UK are limited, and pet ownership specifically in relation to the health of pregnant women and their unborn children, has not been examined.

This study aimed to examine whether pregnant women who owned dogs were more active, particularly through walking, than those pregnant women without pets. The study also assessed whether pregnant women with dogs, were more or less likely to be obese than those without dogs. We hypothesized that those pregnant women who owned dogs would be more active, and have lower weight status.

## Methods

### Selection and description of participants

ALSPAC is a prospective cohort study that has been described in detail elsewhere [26] and on the study website (http://www.bristol.ac.uk/alspac/). In brief, 14 541 pregnant women were recruited, all of whom were resident in Avon, UK, with expected dates of delivery 1st April 1991 to 31st December 1992. Of the initial 14,541 pregnancies, all but 69 had a known birth outcome. Of these 14,472 pregnancies, 195 were twin, three were triplet and one was a quadruplet pregnancy, meaning that there were 14,676 foetuses in the initial ALSPAC sample; 14,062 were live births and 13,988 were alive at 1 year. The twin, triplet and quadruplet children were omitted for this paper, so that the final available dataset comprised 14,273 pregnant mothers of singletons. The majority of mothers were enrolled during pregnancy (from 3 to 41 weeks), and 3% were enrolled after delivery.

### Data collected

ALSPAC has collected data from pregnancy onwards using postal questionnaires, hands-on clinic assessments, biological samples, linkage to routine information, abstraction from medical records and environmental monitoring. Ethical approval for the study was obtained from the ALSPAC Law and Ethics Committee and the Local Research Ethics Committees. Written informed consent was received from all participants.

Maternal activity during pregnancy in this cohort has been described previously [27]. Briefly, at 18 and 32 weeks gestation women were asked whether, at least once a week, they engaged in any regular activity like brisk walking, gardening, housework, jogging, cycling etc, ‘long enough to work up a sweat’. The mother was also asked to indicate the number of hours per week spent in such activity. Women were also asked how long they participated in a variety of activities, including jogging, aerobics, antenatal classes, ‘keep fit’ exercises, yoga, squash, tennis/badminton, swimming, brisk walking, weight training, cycling, and other exercise. Options for response were ≥7 hours, 2–6 hours, <1 hour, or never. Pre-pregnancy BMI of the mother calculated from self-reported height and weight, was used to estimate weight status during pregnancy as few women would be expected to lose weight during pregnancy [28].

Immediately after enrolment in the cohort during pregnancy, the mother was asked ‘do you have any pets?’ and ‘how many of the following pets do you have?’ Pet types prompted included cats, dogs, rabbits, rodents (mice, hamster, gerbil etc), birds (budgerigar, parrot etc) and ‘other’ pets. Pet ownership data have been previously reported [29]. Women who did not indicate that they had had a pet during pregnancy were coded ‘no pets’ and ‘0’ for each pet type.

Data on potential confounding variables were also collected. Socio-demographic factors, such as maternal younger age, not having children and higher levels of education, are known to be associated with higher levels of active living and exercise in women [30]. Similar factors are found to be associated with maternal activity during pregnancy and just prior to pregnancy [31], [32], including in this dataset [27]. A number of factors are also known to be associated with owners of different pet types in this cohort, including age, social class, education, house type, presence of children, number of people in household and previous history of pet ownership, as previously described [29]. Thus, the factors: maternal education, maternal social class, mother worked during pregnancy, maternal age at delivery, number of people in household, previous living children, house type and whether the pregnant woman had pets as a child, were considered as confounding variables.

### Statistical analyses

Given that the duration of strenuous physical activity was reported in hours rather than minutes, we chose a cut-off point (≥3 hours per week) to dichotomize women into two groups. The cut-off point was used to approximate the recommended level of exercise at moderate intensity for 30 minutes or more a day for most days of the week [3], although the ALSPAC question includes vigorous physical activities. Weight status was defined as normal weight (BMI<25), overweight (BMI: 25–29.9), and obese (BMI≥30). Two binary variables were created: normal compared to overweight or obese, and normal compared to obese.

Chi-squared tests and binary or multinominal logistic regression analyses (univariable and multivariable) were used to compare owners and non-owners of different pet types in respect to outcomes of: participates in activity at least once per week (compared to not); equal to or greater than 3 hours of activity a week (compared to less than 3 hours per week); and hours of participation in numerous activity types (never, less than one hour per week, 2–6 hours per week, more than 7 hours per week). Analyses also compared owners and non-owners of different pet types in respect to overweight or obese compared to normal weight (presented); obese compared to normal weight (presented), and obese compared to normal or overweight (not presented but had very similar findings). Confounders were adjusted for in multivariable regression analyses. Participants with missing data for relevant variables were excluded from that analysis. Subgroup analyses were used to investigate the three-way relationships between dog ownership, brisk walking and obesity.

## Results

Of the 14,273 singleton birth mothers in the ALSPAC initial sample, pet ownership data during gestation were reported for 13,215. The study sample characteristics are described in Table 1; 11,466 reported on both pet ownership and activity during pregnancy and thus were available for analysis. During pregnancy, 58.0% (7,670) of pregnant women owned one or more pets; 24.9% had one or more dogs.

**Table 1:** Characteristics (number and percentage) of the pregnant women who submitted pet ownership and activity information (n = 11,466).

Variable	Level	Number	Valid percent
Maternal education	CSE or no qualification (lowest)	2056	19.1
	Vocational	1061	9.9
	O level	3781	35.1
	A level	2472	23.0
	Degree (highest)	1395	13.0
	Missing	701	
Maternal social class	Professional (highest)	535	5.9
	Managerial and technical	2845	31.5
	Skilled: non-manual	3911	43.2
	Skilled: manual	706	7.8
	Partly skilled	858	9.5
	Unskilled (lowest)	190	2.1
	Missing	2421	
Mother worked during pregnancy	No	3061	29.9
	Yes	7166	70.1
	Missing	1239	
Maternal age at delivery	<21 years	671	5.9
	21–30 years	7202	63.0
	>30 years	3560	31.1
	Missing	33	
Number of people in household	2	4426	38.8
	3	4189	36.7
	4	1867	16.4
	5+	935	8.2
	Missing	49	
Has previous living children	No	4913	43.7
	Yes	6333	56.3
	Missing	220	
House type	Detached	1668	14.7
	Semi-detached	3985	35.1
	Terraced	3686	32.4
	Flat/room in someone else's house/other	2023	17.8
	Missing	104	
Mother had pets as a child	No, not at all	821	10.7
	Yes, part of time	3826	45.0
	Yes, always	3864	45.4
	Missing	2955	

### Dog ownership and maternal physical activity during pregnancy

At 18 weeks, 68.7% of 11,875 pregnant women reported engaging in any regular activity at least once a week. Engaging in any regular activity was positively associated with dog ownership only (Table 2). The association was not attenuated (odds ratio 1.27, 95% confidence interval 1.11–1.41, P<0.001), after adjustment for confounders. A similar association was found for the 32 weeks data (not shown).

**Table 2:** Association between maternal activity at 18 weeks and dog ownership during gestation, univariable and multivariable (adjusted).

Outcome (predictor)		n (%)	n (%)	CrudeOR (95%CI)	CrudeP$	Adj* OR (95%CI)	Adj* P$
**Activity at least once a week**		**No**	**Yes**				
Dog present	No	2813(32.5)	5833(67.5)	1		1	
	Yes	797(28.3)	2023(71.7)	1.22(1.11–1.34)	<0.001	1.27(1.11–1.44)	**<0.001**
Number of dogs	No dog	2813(32.5)	5833(67.5)	1		1	
	Single dog	608(27.8)	1579(72.2)	1.25(1.13–1.39)	<0.001	1.31(1.14–1.51)	**<0.001**
	Multiple dogs	189(29.9)	444(70.1)	1.13(0.95–1.35)	0.17	1.12(0.88–1.43)	0.36
**Hours of activity per week**		**<3**	**≥3**				
Dog present	No	4327(53.0)	3834(47.0)	1		1	
	Yes	1153(43.7)	1486(56.3)	1.45(1.33–1.59)	<0.001	1.53(1.35–1.72)	**<0.001**
Number of dogs	No dog	4327(53.0)	3834(47.0)	1		1	
	Single dog	892(43.6)	1156(56.4)	1.46(1.33–1.61)	<0.001	1.56(1.37–1.78)	**<0.001**
	Multiple dogs	261(44.2)	330(55.8)	1.43(1.21–1.69)	<0.001	1.41(1.12–1.78)	**0.004**

* Adjustment after inclusion of maternal education, maternal social class, mother worked during pregnancy, maternal age at delivery, number of people in household, previous living children, house type and whether mother had pets as a child.

$ Likelihood ratio P-value.

At 18 weeks, pregnant women (n = 11,165) reported a median 2 hours of activity per week (mean 5.2), and 49.4% reported ≥3 hours of activity per week. Analysis of the hours of physical activity per week by dog ownership showed a similar relationship to above (Table 2), with those owning a dog being 1.53 times more likely to achieve ≥3 hours a week (1.35–1.72, P<0.001) than those without a dog. There was no evidence that owning multiple dogs, instead of a single dog, was associated with higher reported physical activity, before or after adjustment. In fact, median hours per week spent in activity were 2 for those with no dog, 4 for those with a single dog, and 3 for those with multiple dogs (Kruskal-Wallis P<0.001).

Brisk walking was the only activity type associated with dog ownership after adjustment for other factors. Thus, we present here only the association between dog ownership and brisk walking. Those with dogs were less likely to walk for <1 hour a week (0.78, 0.66–0.93), but more likely to walk 2 to 6 hours per week (1.43, 1.23–1.67) or >7 hours per week (1.80, 1.43–2.27), than never walk. More dog owners reported brisk walking for >7 hours per week compared to those who did not own a dog (11.8% versus 8.5%). However, 693 dog owning women reported never going for a brisk walk, and as a percentage this was the same as for those without dogs (25%).

### Dog ownership and maternal weight status

From n = 12,254 individuals with reported height and weight, 5.1% of pregnant women were classified as obese and 19.1% as overweight or obese. On univariable analysis, dog ownership was associated with maternal overweight or obese; however, after adjusting for confounding factors, the association did not remain (Table 3). Dog ownership was also associated with maternal obesity but again no association remained after adjustment. On univariable analysis, there was a trend for increasing likelihood for pregnant women with multiple dogs to be overweight or obese compared to those with single dogs and no dogs, but there were no differences after adjustment.

**Table 3:** Association between maternal pre-pregnancy BMI and dog ownership during gestation, univariable and multivariable (adjusted).

Outcome (predictor)		n (%)	n (%)	CrudeOR (95%CI)	CrudeP$	Adj* OR (95%CI)	Adj* P$
**Maternal overweight or obese**		**Normal**	**Ovw/Ob**				
Dog present	No	7336(81.6)	1657(18.4)	1		1	
	Yes	2328(79.0)	618(21.0)	1.18(1.06–1.30)	0.002	1.07(0.93–1.24)	0.46
Number of dogs	No dog	7336(81.6)	1657(18.4)	1		1	
	Single dog	1801(79.4)	468(20.6)	1.15(1.03–1.29)	0.02	1.07(0.91–1.25)	0.43
	Multiple dogs	527(77.8)	150(22.2)	1.26(1.04–1.52)	0.02	1.11(0.84–1.46)	0.48
**Maternal obesity**		**Normal**	**Obese**				
Dog present	No	7336(94.5)	429(5.5)	1		1	
	Yes	2328(92.9)	179(7.1)	1.31(1.10–1.57)	0.003	0.97(0.74–1.27)	0.82
Number of dogs	No dog	7336(94.5)	429(5.5)	1		1	
	Single dog	1801(93.3)	129(6.7)	1.22(1.00–1.50)	0.05	0.88(0.65–1.19)	0.39
	Multiple dogs	527(91.3)	50(8.7)	1.62(1.19–2.20)	0.002	1.33(0.84–2.10)	0.23

* Maternal overweight/obesity adjustment for maternal education, maternal social class, mother worked during pregnancy, maternal age at delivery, previous living children, number of people in household, house type, mother had pets as a child.

$ Likelihood ratio P-value.

### Relationship between dog ownership, brisk walking and obesity

Mothers who walked 2 or more hours per week were less likely to be obese than those who walked less than 2 hours a week (after adjustment 0.70, 95% 0.56–0.87, P = 0.001); thus, physical activity through brisk walking appeared to impact weight status. The effect of walking on obesity risk was different between dog owners and non-dog owners: there was no evidence that walking for 2 or more hours a week (compared to less than 2 hours per week) was associated with a reduced odds of obesity in dog owners (0.84, 0.55–1.28, P = 0.41); however, amongst non-dog owners, increased walking was associated with reduced risk of obesity (0.65, 0.50–0.85, P = 0.001). The effect of dog ownership on obesity risk was similar whether the mother walked 2 or more hours per week or less than 2 hours per week (respectively, 1.28, 0.89–1.86, P = 0.19; and 1.12, 0.80–1.56, P = 0.51). Five percent of the dog owners who walked over 2 hours per week were obese, compared with 7% of the dog owners who walked less than 2 hours per week. There was also no difference in the percentage of obesity in mothers who had a dog and walked over 2 hours per week (6%) compared with mothers who did not have a dog (6%). The effect of dog ownership on walking was different in normal weight or overweight mothers compared to obese mothers: in obese mothers there was only weak evidence of an association between dog ownership and walking 2 or more hours per week (1.62, 0.97–2.69, P = 0.07); whereas in overweight or normal weight mothers, dog ownership was positively associated with walking 2 or more hours per week (respectively 1.60, 1.21–2.12, P = 0.001; and 1.53, 1.36–1.72, P<0.001). However, these differences could be due to the smaller sample size of obese mothers meaning decreased power to detect an association.

### Sensitivity analyses – other pet types

During pregnancy, 29.5% of women had one or more cats, 8.7% rabbits, 6.1% rodents, and 7.8% birds. Those who lived with rabbits were also more likely to achieve 3 hours of activity per week (after adjustment 1.29, 1.07–1.55, P = 0.01) and this appeared to be independent of any confounding by dog ownership (data not shown). However, rabbit ownership did not appear to be associated with any particular activity type (data not shown).

On univariable analysis, pet ownership was associated with maternal overweight or obese (for cat, rabbit bird or other pet). However, after adjusting for confounding factors, the association remained only for bird ownership (OR = 1.55, 95%CI = 1.25–1.93, P<0.001). Pet ownership was associated with increased maternal obesity (for cat, dog, bird or other pet, but not rabbit) but, after adjustment, no associations remained except for a weak and attenuated association with cat ownership (OR = 1.27, 95%CI = 1.00–1.62, P = 0.05).

## Discussion

This is the first study to examine the association between pet ownership and physical activity or weight status in pregnant women, albeit using a dataset collected 20 years ago. Dog ownership was associated with an increased (1.5 times) likelihood of undertaking at least 3 hours per week of activity ‘enough to work up a sweat’. Dog owners showed increased levels of brisk walking, but not other types of activity, thus the specificity of the finding makes it more likely that the association is causal. In addition, the trend of increasing likelihood of dog ownership with higher levels of activity and more hours of brisk walking per week also suggests a real effect of owning a dog. Surprisingly, dog ownership appeared at first to be associated with obesity, but this was due to confounding by sociodemographic factors related to both dog ownership and risk factors for obesity. From exploratory analysis (data not shown) this seemed to be mainly due to the variable ‘social class’. Thus, certain family types are more likely to own dogs, and also tend to be overweight or obese prospective mothers, similar to findings for childhood obesity in the same cohort [33].

We conducted sensitivity analyses in the form of testing the effects of ownership of other pet types. There was some evidence to suggest that rabbit ownership was also associated with increased activity. However, rabbit owners did not show increases in any particular activity type, suggesting that this apparent association may be due to an unmeasured confounding variable or a chance finding due to multiple testing. In addition, there was some suggestion that bird ownership was associated with overweight, but not obese women; however, this requires confirmation in other studies because the association was not pre-specified and may, therefore, be a chance finding. We conclude that our findings of associations between dog ownership and walking are specific, supporting the idea that the association may be causal.

The findings from this study are representative of only one population of pregnant women in the UK, in the early 1990s when obesity prevalence was lower, and thus may not be generalisable to other geographical areas or time periods. Health advice given to pregnant women now may be different from that given twenty years ago, demonstrated by the 2009 update to the American Institute of Medicine guidelines on weight management in pregnancy originally published in 1990 [34]. It is possible that twenty years ago, women were advised, either by health professionals or by friends and family, to partake in less physical activity. However, besides a slight under-representation of ethnic minority groups, the baseline ALSPAC cohort was broadly representative of the UK population at the time [26] and provides new original information relating to the activity of pregnant women in relation to dog ownership, of which the prevalence has remained approximately the same [35].

Both the outcome measures (parental weight and height, maternal activity) and the predictor variables of interest (pet ownership) were self-reported. However, self-reported weight and height are generally thought to be reliable in younger adults [36] and, even if BMI was underreported, this would likely lead to under- rather than overestimation of effect sizes [5]. Self-reported physical activity is instead more likely to have been over-reported. It is unlikely that pet ownership was under- or over-reported, since not only is it difficult to see a meaningful reason not to report this accurately, but the likelihood of recall bias was low because the data were collected at the time rather than retrospectively. Nonetheless, given that some of the findings were based on small numbers and multiple testing, these findings should be viewed as preliminary and require further investigation. It is possible that dog walkers are more likely to recall walking because this is an activity specifically focused around dogs and walking, in contrast to walking for other reasons (e.g. to the shops). Even if dog owners are explicitly more active, the direction of the relationship cannot be determined here due to the cross-sectional nature; it may be that more active people who enjoy walking are more likely to acquire dogs, rather than dogs making people more active *per se*.

Associations between increased physical activity of adults and pet ownership, usually dogs, have been reported in a number of other studies [18], [19], [20], [21], [22], [23], [24], [25]. Associations between pet ownership and, increased BMI have been previously reported [37], [38]. However, the strength of our study is that we controlled for a number of other socioeconomic and demographic potential confounders known to be related both to health behaviours and ownership of different pet types. We also analysed these data by individual pet type, showing that these potential relationships are specific and likely due to the ownership of dogs. In addition, our study is the first to our knowledge to examine these behaviours in relation to pet ownership in pregnant women. Higher levels of physical activity of dog walkers have been reported in other studies through the use of objective measures such as accelerometry [21], [25]; since these are more reliable than self reports, they do suggest that the reported higher activity of pregnant dog owners in our study may be meaningful. Owners of a single dog spent a median of 2 extra hours per week in activity, equivalent to a 30 min dog walk, four times a week. Although our study did not report actual dog walking prevalence, this estimation concurs with previously reported patterns of dog walking [39], [40]. In our study, those with multiple dogs reported less walking than those with single dogs, but more than those with no dog, thus owning more dogs is not likely to increase walking behaviour. This concurs with other research suggesting that ‘regular’, as opposed to ‘seldom’, dog walking behaviour appears to be related to the dog providing support and motivation to walk, regardless of other potential factors such as size of dog or number of dogs [39].

Given that we found increased activity in dog walkers, we might also expect to find decreased levels of obesity in dog owners. However, there was little evidence in our study for an association between dog ownership and obesity and, in any case, the direction of any effect was in the direction of increased obesity in dog owners rather than decreased. There are three potential explanations for our findings that dog ownership is associated with increased activity but not decreased weight status. The first theory is that women of child-bearing age with high weight status acquire dogs in order to increase their activity levels; a second possibility is that physical activity attributable to dog ownership (i.e. walking) may not be intense enough to influence weight status in females of child-bearing age; finally it is possible that walking a dog does contribute to decreased BMI, but is masked at a population level by the number of dog owners that do not walk their dog and have increased weight status.

The fact that owners of a single dog, on average, spent only 2 extra hours per week in activity compared to those with no dog, would support the second or third theory. However, our other findings suggest that the second theory is most plausible, namely that the activity of dog walking is not sufficient to influence weight status. Increased walking was associated with decreased odds of obesity in non-dog owners, but not dog owners, suggesting that other factors may have more influence on obesity than walking with a dog, or that the type of walking done with dogs is not as ‘active’ as the walking done without a dog. We also tested the hypothesis that dog owners that walked more would be less likely to be obese than dog owners that walked less (which would support theory 3), but found no evidence for this. We also found only borderline evidence that dog ownership was associated with increased walking in obese mothers, but there was evidence of an association between dog ownership and increased walking in normal weight or overweight mothers. This suggests that obese mothers may be less likely to walk with their dogs than those of lower weight status, or our non-significant finding may be due to the smaller sample of obese mothers. It certainly does not support theory 1. However our analyses must be interpreted with caution as we do not know how much of the reported walking was actually done with the dog. In contrast to our findings, a previous US study showed that those who walked their dogs were more active, and had lower weight status, than those who had a dog but did not walk it [25]; supporting theory 3.

Increased exercise has other potential beneficial health impacts besides just obesity. The main reasons reported by pregnant women for not exercising are feeling unwell or tired, being too busy, or exercise being uncomfortable in late pregnancy [41] and low intensity exercise is perceived as safest and vigorous exercise unsafe [41]. Walking is a readily available, cheap, and recommended exercise activity for many pregnant women, and walking dogs may be underutilised considering the effect sizes seen in our study.

Future research should confirm activity levels in dog owning and non-dog owning pregnant women in a more recent dataset, and using an objective measure such as accelerometry. Studies of activity levels of pregnant women should include specific questions concerning dog walking and her relationship with the dog, such as those contained in the Dogs And Physical Activity (DAPA) tool [42], so that health benefits of dog ownership can be better characterised. In terms for policy and practice, one implication of this research might be to encourage dog ownership in pregnant women, but to do this without due consideration of the suitability of the situation for dog acquisition and welfare of the dog, would be both unethical and unrealistic. More importantly, the potential intervention here is to encourage people who already own dogs to be more physically active with the dogs that they already have. The reasons why some owners, pregnant or not, do not walk their dogs regularly, is still unclear, and requires further research. Occupational and recreational activity is known to decline during pregnancy, in contrast to domestic activity levels, which remain similar [43]. Whether participation in dog walking also declines during pregnancy is not currently known, and may depend on whether it is considered a leisure pursuit or an essential component of general domestic activity. This could be further elucidated in studies that compared activity levels and participation in dog walking, before, during and after pregnancy. It would also be useful to examine the influence of dog ownership and dog walking on weight gain during pregnancy. As in many health related issues where identifying causation is difficult, longitudinal study designs concerning pet ownership, obesity, and activity, may provide fresh insights. Randomised controlled trials testing encouragement of dog walking during pregnancy may also be feasible.

In conclusion, pregnant women who owned dogs were more active through walking than those who did not own dogs, but effect sizes were not large, and this did not influence weight status. These findings are similar to those from other population groups. Considering that physical activity in pregnant women has many health implications, including the problems that lead from obesity, and walking is considered a low risk exercise, participation of pregnant women in dog walking activities requires further examination.
